# Sensitivity to ALK-Directed Therapy in Osteosarcoma With an Acquired *ALK* Rearrangement

**DOI:** 10.1200/PO.23.00287

**Published:** 2023-12-14

**Authors:** Zehra Ordulu, Peter Giunta, Wei-Ting Hung, Yin P. Hung, Judit Simon, Florian J. Fintelmann, Jochen K. Lennerz, Kamila Naxerova, Gregory M. Cote

**Affiliations:** ^1^ Department of Pathology, Immunology and Laboratory Medicine, University of Florida, Gainesville, FL; ^2^ Center for Systems Biology, Massachusetts General Hospital and Harvard Medical School, Boston, MA; ^3^ Graduate Institute of Medical Genomics and Proteomics, College of Medicine, National Taiwan University, Taipei, Taiwan; ^4^ Department of Pathology, Massachusetts General Hospital and Harvard Medical School, Boston, MA; ^5^ Department of Radiology, Division of Thoracic Imaging and Intervention, Massachusetts General Hospital and Harvard Medical School, Boston, MA; ^6^ Department of Internal Medicine, Division of Hematology/Oncology, Massachusetts General Hospital and Harvard Medical School, Boston, MA

## Abstract

**Figure:**
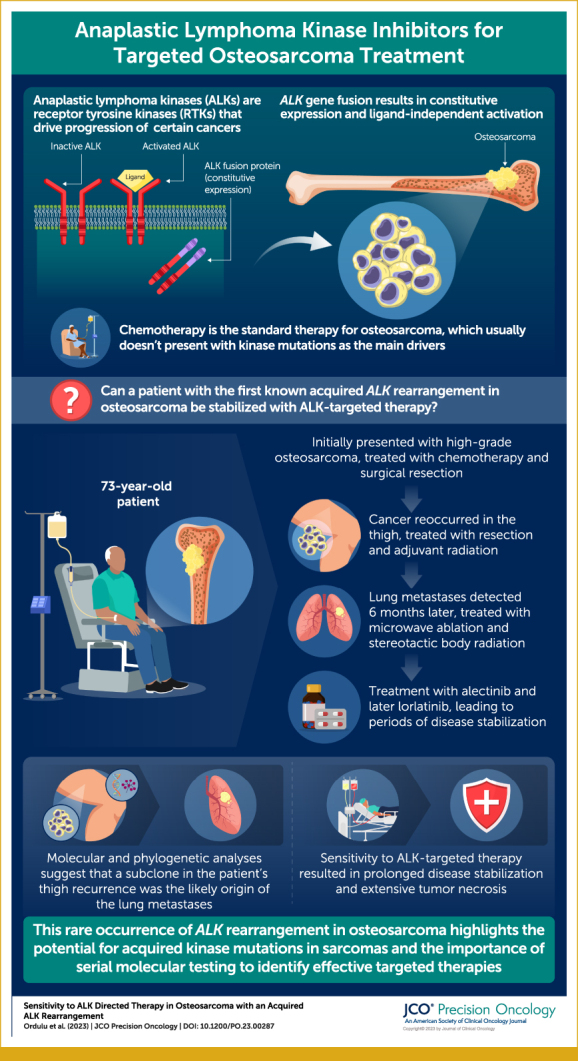

## Background

Sarcomas are clinically, histologically, and molecularly heterogeneous, with at least 70 distinct subtypes occurring in bone and soft tissues.^1^ Molecular alterations most commonly involve tumor suppressors (eg, cell cycle control genes, *TP53*), DNA damage response genes, epigenetic regulators, and, rarely, receptor tyrosine kinases (RTK).^2,3^ The prevalence of a given class of alterations varies widely among histologic types. High-grade osteosarcoma is a malignant tumor of bone with diverse genomics, including mutations in *TP53*, *RB1*, *FGFR1*, *IGF1R,* and DNA structural alterations.^4-7^ In most sarcomas, including osteosarcoma, chemotherapy has traditionally been the backbone of therapy as there is an absence of classic kinase alterations as the main drivers (with exceptions such as GI stromal tumor [GIST], dermatofibrosarcoma protuberans, and inflammatory myofibroblastic tumor [IMT]).

Anaplastic lymphoma kinase (ALK) is an RTK and an oncogenic driver in non–small-cell lung cancer (NSCLC), anaplastic large-cell lymphoma, and IMT, among other tumors. Constitutive expression, most commonly through gene fusions, leads to ligand-independent activation and oncogene addiction. This finding has led to the development of multiple tyrosine kinase inhibitors now approved for *ALK*-rearranged NSCLC, with increasing evidence supporting the use of ALK inhibitors for other ALK-dependent tumor subtypes.^8-11^

Herein, we report a 73-year-old man with an acquired *ALK*-rearranged osteosarcoma. With targeted therapy, the patient showed prolonged disease stabilization of his previously progressing tumor. Phylogenetic analyses revealed a complex clonal relationship among the primary tumor, recurrences, and metastases.

## Case

### Clinical History

**FIG 1.**
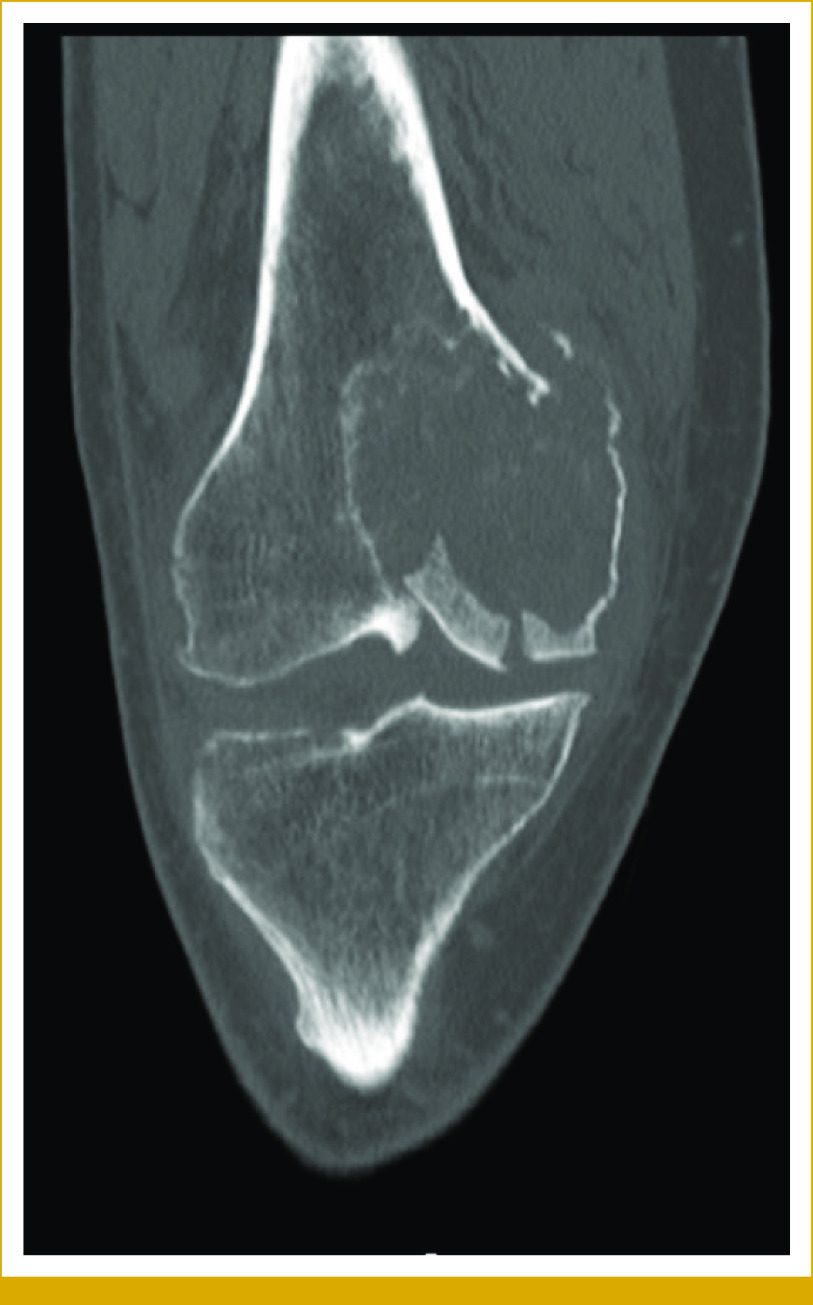
Coronal computed tomography image of a lytic lesion centered in the medial femoral condyle corresponding to the primary osteosarcoma at presentation. Note the presence of a pathologic fracture.

**FIG 2.**
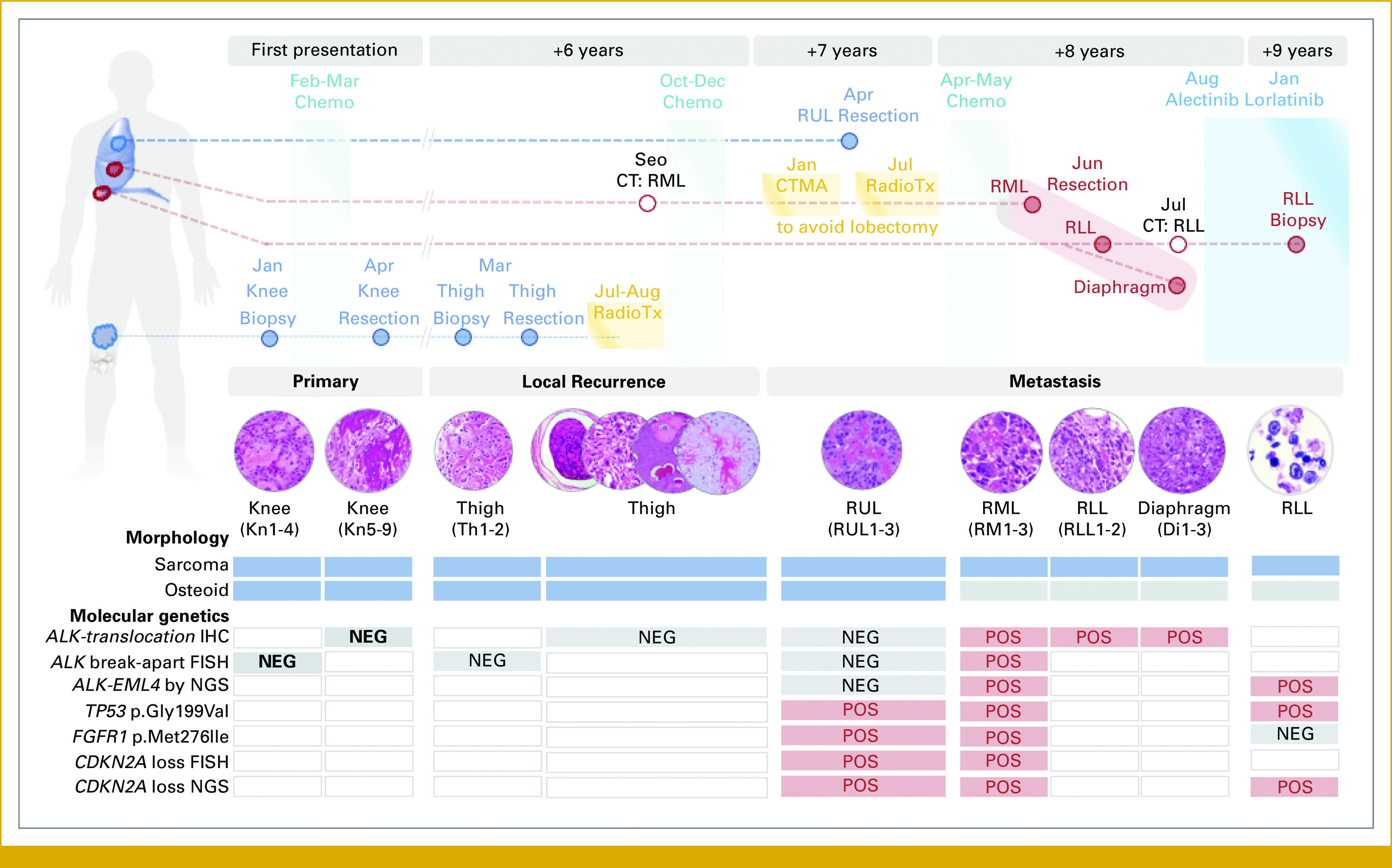
Clinical timeline, tumor morphology, and molecular genotype. Note the lack of overt osteoid morphology coincides with emergence of the *ALK* rearrangement seen in the recent recurrences. The designations in parenthesis are for comparison to Figure 5. Chemo, chemotherapy; CTMA, CT microwave ablation; Di, diaphragm; empty box, not tested; Kn, knee; neg, negative; pos, positive; RadioTx, radiotherapy; RLL, right lower lobe; RML, right middle lobe; RUL, right upper lobe; Th, thigh.

**FIG 3.**
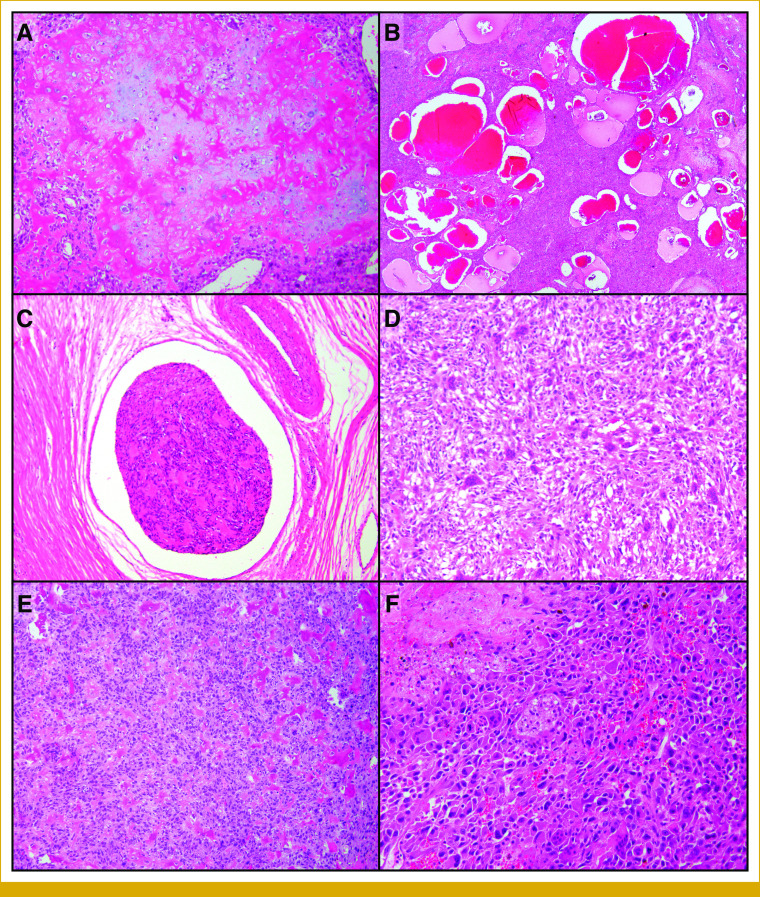
Representative histopathologic images are as follows: (A-D) thigh recurrence at 6 years (*ALK* rearrangement negative) showing (A) chondroblastic features with a chondroid matrix and variable cellularity, (B) telangiectatic features with lakes of blood admixed with malignant cells, (C) prototypical high-grade osteosarcoma with tumor cells associated with osteoid and woven bone, and (D) giant cell–rich morphologies. (E) Right upper lobe metastasis at 7 years (*ALK* rearrangement–negative) with prototypical high-grade osteosarcomatous morphology. (F) Right middle lobe metastasis at 8 years (*ALK* rearrangement positive) showing high-grade pleomorphic histology, with pleomorphic tumor cells, and scattered multinucleated giant cells without definitive osteoid or woven bone deposition. Although the thigh recurrence showed a variety of morphologies, the lymphovascular invasion showed the prototypical osteosarcoma morphology as depicted by (C).

The patient first presented at age 63 years with a 6.6-cm right medial condyle lytic lesion and an associated pathologic fracture (Fig 1). Biopsy revealed high-grade osteosarcoma, and staging studies were free of metastases. He initiated chemotherapy with methotrexate, doxorubicin, and cisplatin. After two cycles, the lesion was resected and found to be 20% necrotic. After surgery, he received adjuvant ifosfamide and etoposide. Six years later, biopsy confirmed recurrence in the soft tissue of the thigh. He underwent resection followed by adjuvant radiation. Unfortunately, within 6 months, he developed lung metastases. Attempts were made to control metastases with ablations, stereotactic body radiation (SBRT), chemotherapy, and resection. At year 7 after first presentation, there was further progression. He was a life-long nonsmoker with no other significant medical history. The longitudinal timeline, including systemic therapy, tumor morphology, and molecular genotype, is shown in Figure 2. Detailed histopathologic images are shown Figure 3.

**FIG 4.**
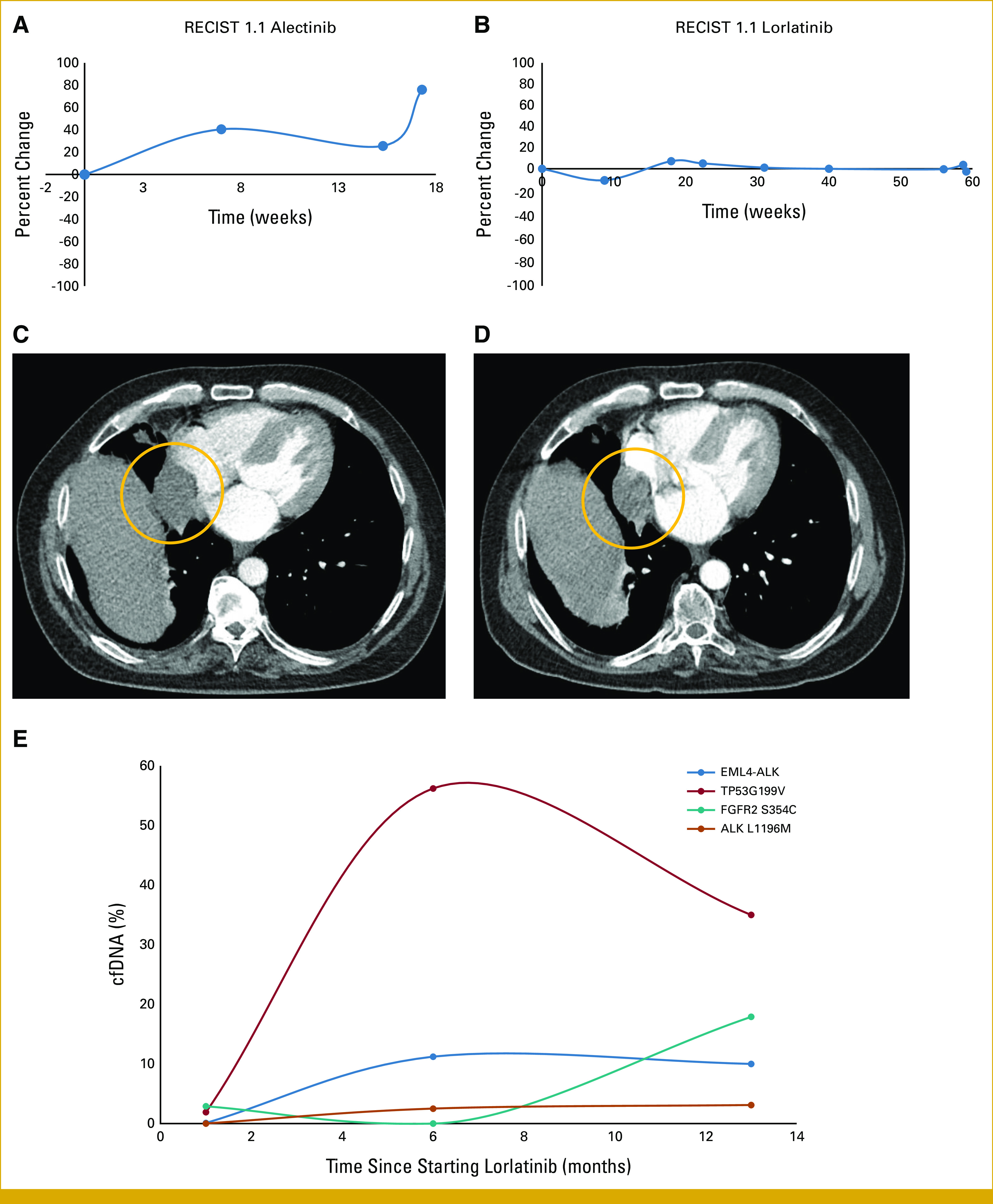
RECIST 1.1 measurements of the lung metastases during ALK-targeted therapy. (A) After initial growth, the target tumors stabilized on alectinib for approximately 10 weeks, before progression. (B) Subsequently, the patient received lorlatinib and had stable disease for nearly 60 weeks. Axial CT image of a lung metastases (circle) (C) before and (D) after 2 months on lorlatinib. (E) cfDNA alteration % change while on lorlatinib. cfDNA, cell-free DNA.

As discussed below (molecular profiling), the *ALK* fusion was first detected in a right middle lobe (RML) lung metastasis, which received microwave ablation and SBRT before resection, unlike previous sites. After further progression, the patient was initiated on off-label alectinib. There was initial tumor growth (+40.5% by Response Evaluation Criteria in Solid Tumors [RECIST] 1.1; Fig 4), followed by disease stabilization on subsequent scans (+26.6% from baseline). After 5 months of alectinib, there was progression and he was transitioned to lorlatinib. Serial imaging confirmed stable disease for over 400 days (best response, stable disease [–11% by RECIST 1.1]; Fig 4). The patient continued lorlatinib with clinical benefit for 14 months, at which point he developed a wound infection in the right knee. This was managed with antibiotics and surgical debridement. The patient was admitted to his local hospital with sepsis. Despite maximal medical intervention, his health condition continued to decline. Ultimately, the decision was made to transition to comfort measures, and he passed away peacefully with his family at the bedside.

### Histology

The primary right femur osteosarcoma was characterized by clusters and sheets of tumor cells associated with osteoid and woven bone, prototypical of a high-grade osteosarcoma. A thigh recurrence at 6 years displayed intermingling of conventional (osteoblastic), chondroblastic, giant cell–rich, and telangiectatic (cystic) areas, representing a recurrent high-grade osteosarcoma. Resection of a right upper lobe (RUL) metastasis at year 7 showed high-grade osteosarcoma with osteoid and woven bone. The resected metastases in the RML, right lower lobe (RLL), and diaphragm at year 8 displayed high-grade pleomorphic histology, with sheets of hyperchromatic tumor cells, extensive necrosis, and scattered multinucleated giant cells. Although there were no overt osteoid or woven bone deposition, that tumor was considered compatible with metastatic/recurrent osteosarcoma because of its histologic similarities to those in the previous specimens, and the subsequent molecular workup. Moreover, there were no distinctive histologic features in these specimens that would suggest to the pathologist to evaluate for an acquired *ALK* fusion event (Fig 3).

### Molecular Genetic Profiling

The initial clinical molecular profiling was performed on the RML metastasis at year 8 after the primary diagnosis (Fig 2). Comprehensive genotyping using RNAseq showed an *EML4*::*ALK* fusion (confirmed by immunohistochemistry [IHC] and florescence in situ hybridization [FISH]). In addition, there were *TP53*^*G199V*^ and *FGFR1*^*M276I*^ mutations, as well as *CDKN2A* loss by DNAseq, the latter also confirmed by FISH. The presence of *ALK* fusion was confirmed in the concurrent RLL and diaphragm metastases by IHC. The follow-up cytology specimen from RLL a year later had overlapping alterations of *ALK*, *TP53*, and *CDKN2A*, and lacked the *FGFR1* mutation.

The primary tumor and thigh recurrence at 6 years were negative for *ALK* rearrangement by FISH or IHC (next-generation sequencing [NGS] failed because of decalcification). The RUL tumor with classic osteosarcoma morphology at year 7 had overlapping alterations with the previously described RML tumor, including *TP53* and *FGFR1* mutations, as well as *CDKN2A* loss, and it also did not harbor the *ALK* fusion (confirmed by RNAseq, FISH, and IHC).

Clinical circulating cell-free DNA (cfDNA) testing^12,13^ was performed at months 1, 6, and 13 after starting lorlatinib. First cfDNA showed the *EML4*::*ALK* fusion, *TP53*^*G199V*^, and *FGFR2*^S354C^. At 6 months, cfDNA showed the *EML4*:*ALK* fusion, an *ALK*^L1196M^ mutation (known to confer resistance to crizotinib/alectinib), and the *TP53*^*G199V*^. The *FGFR2*^*S354C*^ was not detectable. At 13 months, cfDNA showed the *ALK* fusion, the *ALK*^L1196M^ resistance mutation, *TP53*^*G199V*^, *FGFR2* ^S354C^, and loss of heterozygosity in *BRCA2*/*ATM*/*PALB2*. From 6 through 13 months of lorlatinib, there was stabilization of the *ALK/EML4::*ALK allele fractions and decrease in the dominant *TP53* allele fraction (Fig 4E).

### Phylogenetic Analysis

**FIG 5.**
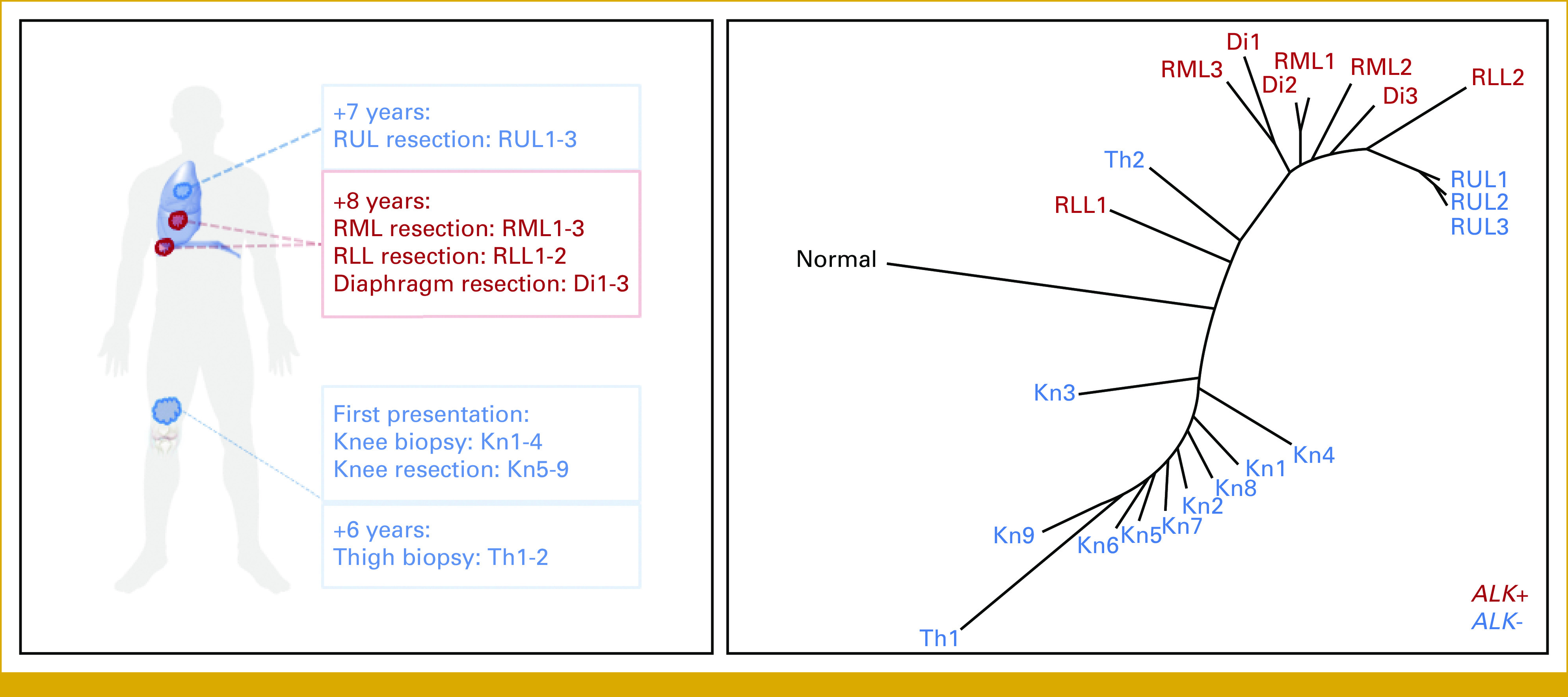
Polyguanine-based phylogenetic tree (right) showing the clonal relationship between the primary osteosarcoma (Kn1-Kn9), local recurrence (Th1-2), and metastatic RUL tumor (RUL1-3) without the *ALK* rearrangement, as well as metastatic concurrent RML (RML1-3), RLL (RLL1-2), and Di (Di1-3) tumors (left) with the *ALK* rearrangement. Di, diaphragm; RML, right middle lobe; RLL, right lower lobe; RUL, right upper lobe.

Phylogenetic reconstruction of the tumor evolutionary history showed shared variants in multiple polyguanine repeats in independent samples from the primary knee tumor, thigh recurrence, and metastases to the lung and diaphragm, confirming that all tumors were clonally related. On the phylogenetic tree (Fig 5), samples segregated along two main branches: one branch comprised all the primary tumor samples (Kn1-9) and one region from the thigh recurrence (Th1); the other branch contained the second thigh recurrence region (Th2) and all thoracic metastases. These results are compatible with a scenario in which a localized subclone in the year 6 thigh recurrence seeded the lung metastases. Although the putative metastasis-seeding region of the thigh recurrence (Th2) and RUL metastasis (RUL1-3) at 7 years were negative for the *ALK* fusion, all other lung metastases (RML1-3, RLL1-2), as well as the diaphragm metastases at 8 years (Di1-3), were positive. This indicates a descendant of the putative metastatic subclone resident in Th2 acquired the *ALK* fusion, and that some metastases (RLL, RML, and Di) were seeded after this acquisition, while other metastases (RUL) were seeded independently (either earlier in time or by a parallel descendant of the Th2 subclone).

### Autopsy Findings

Autopsy was performed after consent. There was a large RLL metastasis with extensive necrosis (>90%), along with inferior vena cava tumor thrombus. Microscopically, tumor cells appeared spindled-pleomorphic and showed diffuse strong ALK immunoreactivity, consistent with *ALK*-rearranged high-grade osteosarcoma. Bilateral patchy acute pneumonia and infection of right leg soft tissue were identified. The cause of death was metastatic high-grade osteosarcoma complicated by infection.

Methods are discussed in the Data Supplement (Appendix 1).

## Discussion

Here, to our knowledge, we document the first known acquired *ALK* rearrangement in osteosarcoma. Reconstructing the timeline and integration of morphologic, molecular, and phylogenetic findings confirmed shared clonal origin between the primary and the recurrent/metastatic tumors despite the morphologic and molecular variety. The sensitivity to ALK-targeted therapy was demonstrated with prolonged disease stabilization of the previously rapidly progressing disease and extensive tumor necrosis in the autopsy. Finally, the changes in cfDNA allele fraction and emergence of a resistance mutation confirm antitumor pressure. Although this is a rare occurrence, our report highlights the potential for acquired kinase mutations in heavily treated malignancies and suggest ALK-directed therapy may be an option for acquired *ALK* rearrangements.

In the present case, the *ALK* rearrangement first emerged in lung metastases 8 years after resection of the primary. Phylogenetic analysis revealed that a subclone in the patient's thigh recurrence was the likely origin of the lung metastases. Although the patient received multiple cycles of chemotherapy and radiotherapy, interestingly, the first *ALK*-rearranged tumors developed in the setting of direct tumor exposure to microwave ablation and radiation, which may be suggestive of a thermal and/or radiation-related event or resistance mechanism. Emergence of *ALK* rearrangements in response to therapy has been reported in lung carcinomas^14,15^; however, the chemotherapy/radiotherapy/ablation status of these tumors was not clearly documented. To our knowledge, this phenomenon of an acquired *ALK* rearrangement in the setting of extensive therapies has not been documented in sarcomas. In addition, there is only a single patient with osteosarcoma in a previous case series of *ALK*-rearranged nonlung solid tumors.^16^ However, the context of primary versus acquired status and the oncogenic role of the *ALK* rearrangement are unclear on the basis of the data provided. Overall, to our knowledge, the findings of our case represent the first actionable *ALK* rearrangement in osteosarcomas, as well as its acquired status, which previously has not been established in sarcomas.

In addition to the molecular evolution of this osteosarcoma, there were morphologic changes over time. The resected RML metastasis, the first tumor where we detected the *ALK* rearrangement, was morphologically distinct (ie, an absence of osteoid formation) from the previous tumors, including an earlier lung metastasis with typical osteosarcoma morphology. This observation raised the possibility of a new primary tumor as a clinical consideration during the patient's care. This was ruled out by integrating phylogenetic assessment into the NGS findings (Figs 2 and 5). Although the phylogenetic evaluation is not a clinical assay, in the future, it may assist in understanding the temporal evaluation of genetic lesions, both for precursor lesions^17^ and subsequent metastases.^18^ These findings highlight that relying on morphologic features alone would not be adequate to characterize tumors in scenarios such as this case. Overall, our data emphasize the importance of multisite sampling at different stages of disease progression while integrating molecular data to guide therapeutic considerations. This integrative approach will aid the pathology and oncology teams in determining if there is a second independent primary tumor and identify actionable alterations.

In conclusion, this *ALK* rearrangement in an osteosarcoma is a rare finding that demonstrates acquired RTK alterations are possible in sarcoma. This supports the concept of serial molecular testing through the course of treatment where targeted therapy may be a therapeutic option. Finally, phylogenetic analysis can provide valuable information on the clonal relationship between primary tumors and metastases for morphologically and molecularly challenging cases, which may have implications for treatment decisions.

## References

[1] WHO Classification of Tumours Editorial Board: WHO classification of tumours series: Soft tissue and bone tumours (ed 5). Lyon, France, International Agency for Research on Cancer, 2020

[2] Nacev BA, Sanchez-Vega F, Smith SA, et al: Clinical sequencing of soft tissue and bone sarcomas delineates diverse genomic landscapes and potential therapeutic targets. Nat Commun 13:3405, 2022 35705560 10.1038/s41467-022-30453-x PMC9200818

[3] Cote GM, He J, Choy E: Next-generation sequencing for patients with sarcoma: A single center experience. Oncologist 23:234-242, 2018 28860410 10.1634/theoncologist.2017-0290 PMC5813739

[4] Fernanda Amary M, Ye H, Berisha F, et al: Fibroblastic growth factor receptor 1 amplification in osteosarcoma is associated with poor response to neo-adjuvant chemotherapy. Cancer Med 3:980-987, 2014 24861215 10.1002/cam4.268 PMC4303166

[5] Behjati S, Tarpey PS, Haase K, et al: Recurrent mutation of IGF signalling genes and distinct patterns of genomic rearrangement in osteosarcoma. Nat Commun 8:15936, 2017 28643781 10.1038/ncomms15936 PMC5490007

[6] Chen X, Bahrami A, Pappo A, et al: Recurrent somatic structural variations contribute to tumorigenesis in pediatric osteosarcoma. Cell Rep 7:104-112, 2014 24703847 10.1016/j.celrep.2014.03.003 PMC4096827

[7] Mejia-Guerrero S, Quejada M, Gokgoz N, et al: Characterization of the 12q15 MDM2 and 12q13-14 CDK4 amplicons and clinical correlations in osteosarcoma. Genes Chromosomes Cancer 49:518-525, 2010 20196171 10.1002/gcc.20761

[8] Gaule M, Pesoni C, Quinzii A, et al: Exceptional clinical response to alectinib in pancreatic acinar cell carcinoma with a novel ALK-KANK4 gene fusion. JCO Precis Oncol 10.1200/PO.21.00400 10.1200/PO.21.00400 PMC8769132 35005993

[9] Dayyani F, Lee W, Houshyar R, et al: Rapid and deep response to lorlatinib in pancreatic high-grade neuroendocrine carcinoma with a treatment emergent novel KANK1-ALK fusion. JCO Precis Oncol 10.1200/PO.22.00230 10.1200/PO.22.00230 36623237

[10] Gene-Olaciregui N, Perez-Somarriba M, Santa-Maria V, et al: Clinical and molecular evolution of an ALK-driven infant-type hemispheric glioma treated sequentially with second- and third-generation anaplastic lymphoma kinase inhibitors. JCO Precis Oncol 10.1200/PO.22.00547 10.1200/PO.22.00547 36996378

[11] Schoffski P, Sufliarsky J, Gelderblom H, et al: Crizotinib in patients with advanced, inoperable inflammatory myofibroblastic tumours with and without anaplastic lymphoma kinase gene alterations (European Organisation for Research and treatment of cancer 90101 CREATE): A multicentre, single-drug, prospective, non-randomised phase 2 trial. Lancet Respir Med 6:431-441, 2018 29669701 10.1016/S2213-2600(18)30116-4

[12] Odegaard JI, Vincent JJ, Mortimer S, et al: Validation of a plasma-based comprehensive cancer genotyping assay utilizing orthogonal tissue- and plasma-based methodologies. Clin Cancer Res 24:3539-3549, 2018 29691297 10.1158/1078-0432.CCR-17-3831

[13] Zill OA, Banks KC, Fairclough SR, et al: The landscape of actionable genomic alterations in cell-free circulating tumor DNA from 21,807 advanced cancer patients. Clin Cancer Res 24:3528-3538, 2018 29776953 10.1158/1078-0432.CCR-17-3837

[14] Zeng Z, Wang T, He J, et al: ALK-R3HDM1 and EML4-ALK fusion as a mechanism of acquired resistance to gefitinib: A case report and literature review. Front Oncol 12:1010084, 2022 36387181 10.3389/fonc.2022.1010084 PMC9660230

[15] Offin M, Somwar R, Rekhtman N, et al: Acquired ALK and RET gene fusions as mechanisms of resistance to osimertinib in EGFR-mutant lung cancers. JCO Precis Oncol 10.1200/PO.18.00126 10.1200/PO.18.00126 PMC6447364 30957057

[16] Takeyasu Y, Okuma HS, Kojima Y, et al: Impact of ALK inhibitors in patients with ALK-rearranged nonlung solid tumors. JCO Precis Oncol 10.1200/PO.20.00383 10.1200/PO.20.00383 PMC8140781 34036223

[17] Leshchiner I, Mroz EA, Cha J, et al: Inferring early genetic progression in cancers with unobtainable premalignant disease. Nat Cancer 4:550-563, 2023 37081260 10.1038/s43018-023-00533-y PMC10132986

[18] Reiter JG, Hung WT, Lee IH, et al: Lymph node metastases develop through a wider evolutionary bottleneck than distant metastases. Nat Genet 52:692-700, 2020 32451459 10.1038/s41588-020-0633-2 PMC7343611

